# The genome sequence of *Petasites hybridus* (L. G.Gaertn., B.Mey. & Scherb., 1802) (Asterales: Asteraceae)

**DOI:** 10.12688/wellcomeopenres.27231.1

**Published:** 2026-07-29

**Authors:** Markus Ruhsam, Peter M. Hollingsworth, Royal Botanic Garden Edinburgh Genome Acquisition Lab, Plant Genome Sizing Collective, Darwin Tree of Life Barcoding collective, Wellcome Sanger Institute Tree of Life Management, Samples and Laboratory team, Wellcome Sanger Institute Scientific Operations: Sequencing Operations, Wellcome Sanger Institute Tree of Life Core Informatics team, Tree of Life Core Informatics collective, Darwin Tree of Life Consortium

**Affiliations:** 1 Royal Botanic Garden Edinburgh, Edinburgh, Scotland, UK

**Keywords:** Petasites hybridus, Butterbur, genome sequence, chromosomal, Asterales

## Abstract

We present a genome assembly of *Petasites hybridus* (Butterbur; Streptophyta; Magnoliopsida; Asterales; Asteraceae). The assembly consists of two haplotypes with total lengths of 2 802.87 megabases and 2 756.82 megabases. Most of haplotype 1 (95.61%) is scaffolded into 30 chromosomal pseudomolecules. Haplotype 2 was assembled to scaffold level. The mitochondrial sequence has a length of 302.34 kilobases and the plastid genome assembly has a length of 150.22 kilobases. Gene annotation of this assembly on Ensembl identified 41 187 protein-coding genes. This assembly was generated as part of the Darwin Tree of Life project, which produces reference genomes for eukaryotic species found in Britain and Ireland.

## Species taxonomy

Eukaryota; Viridiplantae; Streptophyta; Streptophytina; Embryophyta; Tracheophyta; Euphyllophyta; Spermatophyta; Magnoliopsida; Mesangiospermae; eudicotyledons; Gunneridae; Pentapetalae; asterids; campanulids; Asterales; Asteraceae; Asteroideae; Senecioneae; Tussilagininae; *Petasites*; *Petasites hybridus* (L. G.Gaertn., B.Mey. & Scherb., 1802) (NCBI:txid278673).

## Background

*Petasites hybridus* (commonly known as butterbur) is a large perennial rhizomatous geophyte, widely distributed across temperate Europe and western Asia, typically occurring in nutrient-rich, permanently moist to seasonally inundated habitats such as riverbanks, floodplains, ditches and damp woodland margins ( POWO, 2026; Stroh *et al.*, 2020). Its strong association with riparian systems reflects its ecological preference for high-moisture, high-nutrient environments, where rapid early-season growth allows effective occupation of open space prior to canopy closure in floodplain herbaceous communities.

The species spreads extensively via a persistent rhizome system, enabling extensive clonal growth and the formation of dense, often monospecific stands. This growth form facilitates rapid spatial expansion in suitable habitats and contributes to the species’ highly competitive presence in riparian vegetation. Above-ground shoots emerge very early in spring, producing large leaves that rapidly intercept available light. From a functional ecological perspective, these characteristics align with competitive strategies in resource-rich habitats as described in Grime’s CSR framework, where rapid growth and strong resource pre-emption are typical of species occupying productive, low-stress environments ( Grime, 1977).

Individual *Petasites hybridus* plants appear to be hermaphroditic (i.e., male and female organs are present in the same flower), but are functionally dioecious, as flowers lack the functional reproductive organ of the opposite sex ( Düll & Kutzelnigg, 2022; Haratym & Weryszko-Chmielewska, 2012; Stace, 2019). Species-specific observations indicate that male and female inflorescences differ in floral density and display, and early flowering provides a critical resource for insect pollinators, reinforcing functional separation of the sexes within the reproductive system ( Haratym & Weryszko-Chmielewska, 2012). Experimental evidence from related species in the genus suggests that sterile floral structures may further modulate pollinator behaviour and reproductive success, demonstrating that dioecy in *Petasites* is both structurally and functionally reinforced ( Yu *et al.*, 2011).

*Petasites hybridus* has a long history of medicinal use in Europe, where extracts of the rhizomes and leaves were traditionally employed to treat fever, respiratory ailments and spasmodic disorders ( Aydın *et al.*, 2013). The species contains a range of biologically active sesquiterpene esters, particularly petasin, isopetasin and neopetasin, which are considered the principal pharmacologically active constituents and have been shown to possess anti-inflammatory and antispasmodic properties ( Aydın *et al.*, 2013; Thomet *et al.*, 2001). Standardised butterbur extracts have been investigated for migraine prophylaxis and the treatment of allergic rhinitis, with several clinical studies reporting therapeutic efficacy ( Aydın *et al.*, 2013; Thomet *et al.*, 2001). However, the plant also produces pyrrolizidine alkaloids (PAs), compounds with hepatotoxic and potentially carcinogenic properties, meaning that only PA-free extracts are considered suitable for medicinal use ( Aydın *et al.*, 2013). The coexistence of pharmacologically valuable secondary metabolites and toxic alkaloids has made *P. hybridus* an important model species in phytochemical and medicinal plant research.

The chromosome number of *Petasites hybridus* is consistently reported to be 2 *n* = 60 ( Al-Bermani *et al.*, 1993; Kiehn *et al.*, 1991; Stace, 2019), which is consistent with the genome assembly presented here.

**Figure 1.**
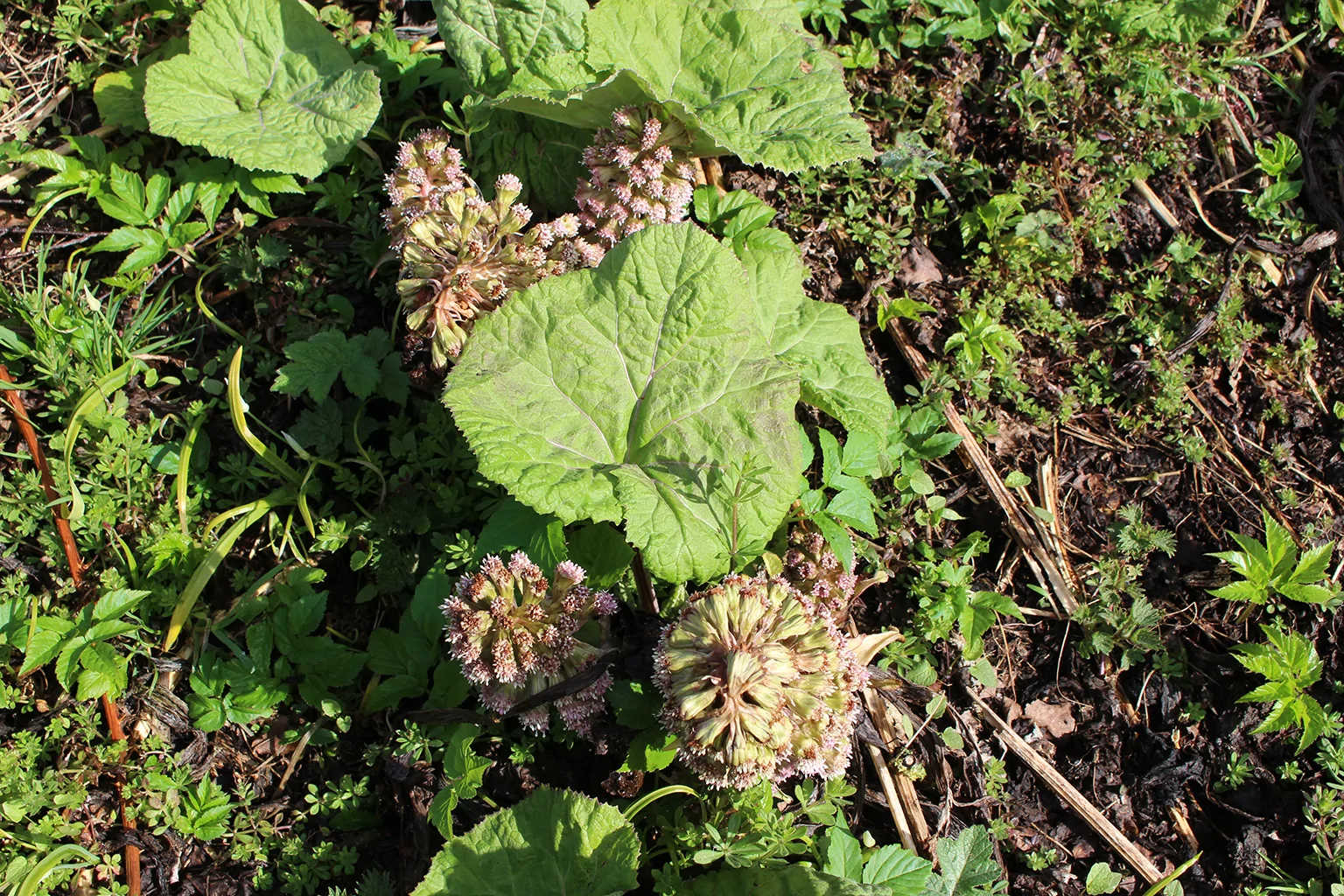
Photograph of the *Petasites hybridus* plant (daPetHybr1) from which samples were taken for genome sequencing.

We present a chromosome-level genome sequence for *Petasites hybridus.* The assembly was produced using the Tree of Life pipeline from a specimen collected in Edinburgh, Scotland, UK ( Figure 1).

## Methods

### Sample acquisition, flow cytometry and DNA barcoding

A specimen of *Petasites hybridus* (specimen ID EDTOL03655, ToLID daPetHybr1; Figure 1) was used for genome sequencing. It was collected from Edinburgh, Hermitage, UK (latitude 55.919, longitude −3.1833) on 2022-04-06. The specimen was collected and identified by Markus Ruhsam (Royal Botanic Garden Edinburgh). The daPetHybr1 specimen was also used for Hi-C and RNA sequencing. The herbarium voucher associated with the sequenced plant is E01152469 and is deposited in the herbarium of RBG Edinburgh (E) <https://data.rbge.org.uk/herb/E01152469>.

The genome size was estimated by flow cytometry following the ‘one-step’ method outlined in Pellicer *et al*. (2021) and using propidium iodide as the fluorochrome. The General Purpose Buffer (GPB) supplemented with 3% PVP was used for isolation of nuclei ( Loureiro *et al.*, 2007), and the internal calibration standard was *Petroselinum crispum* ‘Champion Moss Curled’ with an assumed 1C-value of 2 200 Mb ( Obermayer *et al.*, 2002).

The initial identification was verified by an additional DNA barcoding process according to the framework developed by Twyford *et al*. (2024). Part of the plant specimen was preserved in silica gel desiccant ( Chase & Hills, 1991). DNA extracted from the dried plant was amplified by PCR for standard barcode markers, with the amplicons sequenced and compared to public sequence databases including GenBank and the Barcode of Life Database (BOLD) ( Ratnasingham & Hebert, 2007). Following whole genome sequence generation, the relevant DNA barcode region was also used alongside the initial barcoding data for sample tracking at the WSI ( Twyford *et al.*, 2024). The standard operating procedures for Darwin Tree of Life barcoding are available on protocols.io.

### Nucleic acid extraction

Protocols for high molecular weight (HMW) DNA extraction developed at the Wellcome Sanger Institute (WSI) Tree of Life Core Laboratory are available on protocols.io ( Howard *et al.*, 2025). The daPetHybr1 sample was weighed and triaged to determine the appropriate extraction protocol. For HMW DNA extraction, 68.30 mg of leaf tissue was used. Tissue from the leaf was homogenised by cryogenic disruption using the Covaris cryoPREP® Automated Dry Pulverizer. HMW DNA was extracted using the Automated Plant MagAttract v2 protocol. DNA was sheared into an average fragment size of 12–20 kb following the Megaruptor®3 for LI PacBio protocol. Sheared DNA was purified by manual SPRI (solid-phase reversible immobilisation), using (Pacific Biosciences) AMPure PB beads to eliminate shorter fragments and concentrate the DNA. The concentration of the sheared and purified DNA was assessed using a Nanodrop spectrophotometer and Qubit Fluorometer using the Qubit dsDNA High Sensitivity Assay kit. Fragment size distribution was evaluated by running the sample on the FemtoPulse system. For this sample, the final post-shearing DNA had a Qubit concentration of 7.04 ng/μL and a yield of 319.62 ng, with a fragment size of 13.6 kb. The 260/280 spectrophotometric ratio was 2.01, and the 260/230 ratio was 1.08. The Genomic Quality Number (GQN) was 7.1.

RNA was extracted from leaf tissue of daPetHybr1 in the Tree of Life Laboratory at the WSI using the RNA Extraction: Automated MagMax™ *mir*Vana protocol. The RNA concentration was assessed using a Nanodrop spectrophotometer and a Qubit Fluorometer using the Qubit RNA Broad-Range Assay kit. Analysis of the integrity of the RNA was done using the Agilent RNA 6000 Pico Kit and Eukaryotic Total RNA assay.

### PacBio HiFi library preparation and sequencing

Library preparation and sequencing were performed at the WSI Scientific Operations core. Libraries were prepared using the SMRTbell Prep Kit 3.0 (Pacific Biosciences) according to the manufacturer’s instructions. The kit includes reagents for end repair/A-tailing, adapter ligation, post-ligation SMRTbell bead clean-up, and nuclease treatment. Size selection and clean-up were performed using diluted AMPure PB beads (Pacific Biosciences). DNA concentration was quantified using a Qubit Fluorometer v4.0 (ThermoFisher Scientific) and the Qubit 1X dsDNA HS assay kit. Final library fragment size was assessed with the Agilent Femto Pulse Automated Pulsed Field CE Instrument (Agilent Technologies) using the gDNA 55 kb BAC analysis kit.

The sample was sequenced using the Sequel IIe system (Pacific Biosciences, California, USA). The concentration of the library loaded onto the Sequel IIe was in the range 40–135 pM. SMRT Link software, a PacBio web-based workflow manager, was used to set up and monitor the run, and to perform primary and secondary analysis of the data upon completion.

### Hi-C

***Sample preparation and crosslinking***

Hi-C data were generated from the leaf tissue of daPetHybr1 using the Arima-HiC v2 kit (Arima Genomics). Tissue was finely ground using the Covaris cryoPREP Dry Pulverizer (Covaris), and then subjected to nuclei isolation. Nuclei were isolated using a modified protocol based on the Qiagen QProteome Cell Compartment Kit (Qiagen), in which only the Lysis and CE2 buffers were used, with QIAshredder spin columns. After isolation, nuclei were fixed using formaldehyde to a final concentration of 2% to crosslink the DNA. The crosslinked DNA was then digested and biotinylated according to the manufacturer’s instructions. A clean-up step was performed with SPRIselect beads before library preparation. DNA concentration was quantified using the Qubit Fluorometer v4.0 (Thermo Fisher Scientific) and the Qubit HS Assay Kit, following the manufacturer’s instructions.

***Hi-C library preparation and sequencing***

Biotinylated DNA constructs were fragmented using a Covaris E220 sonicator and size selected to 400–600 bp using SPRISelect beads. DNA was enriched with Arima-HiC v2 kit Enrichment beads. End repair, A-tailing, and adapter ligation were carried out with the NEBNext Ultra II DNA Library Prep Kit (New England Biolabs), following a modified protocol where library preparation occurs while DNA remains bound to the Enrichment beads. Library amplification was performed using KAPA HiFi HotStart mix and a custom Unique Dual Index (UDI) barcode set (Integrated DNA Technologies). Depending on sample concentration and biotinylation percentage determined at the crosslinking stage, libraries were amplified with 10–16 PCR cycles. Post-PCR clean-up was performed with SPRISelect beads. Libraries were quantified using the AccuClear Ultra High Sensitivity dsDNA Standards Assay Kit (Biotium) and a FLUOstar Omega plate reader (BMG Labtech).

Prior to sequencing, libraries were normalised to 10 ng/μL. Normalised libraries were quantified again to create equimolar and/or weighted 2.8 nM pools. Pool concentrations were checked using the Agilent 4200 TapeStation (Agilent) with High Sensitivity D500 reagents before sequencing. Sequencing was performed using paired-end 150 bp reads on the Illumina NovaSeq X.

### RNA library preparation and sequencing

Libraries were prepared using the NEBNext® Ultra II Directional RNA Library Prep Kit for Illumina (New England Biolabs), following the manufacturer’s instructions. Poly(A) mRNA was isolated from 100 ng of total RNA using oligo (dT) beads, converted to cDNA, and uniquely indexed; 14 PCR cycles were performed. Libraries were prepared with an intended fragment size of 100–300 bp. Libraries were quantified, normalised, and pooled equimolarly to a final concentration of 2.8 nM before dilution to 150 pM for loading. Sequencing was carried out on the Illumina NovaSeq X, generating paired-end reads.

### Genome assembly

Prior to assembly of the PacBio HiFi reads, a database of *k*-mer counts ( *k* = 31) was generated from the filtered reads using FastK. GenomeScope2 ( Ranallo-Benavidez *et al.*, 2020) was used to analyse the *k*-mer frequency distributions, providing estimates of genome size, heterozygosity, and repeat content.

The HiFi reads were assembled using Hifiasm in Hi-C phasing mode ( Cheng *et al.*, 2021, 2022), producing two haplotypes. Hi-C reads ( Rao *et al.*, 2014) were mapped to the primary contigs using bwa-mem2 ( Vasimuddin *et al.*, 2019). Contigs were further scaffolded with Hi-C data in YaHS ( Zhou *et al.*, 2023), using the --break option for handling potential misassemblies. The scaffolded assemblies were evaluated using Gfastats ( Formenti *et al.*, 2022), BUSCO ( Manni *et al.*, 2021) and MerquryFK ( Rhie *et al.*, 2020). The organelle genomes were assembled using OATK ( Zhou *et al.*, 2025).

### Assembly curation

The assembly was decontaminated using the Assembly Screen for Cobionts and Contaminants ( ASCC) pipeline. TreeVal was used to generate the flat files and maps for use in curation. Manual curation was conducted primarily in PretextView and HiGlass ( Kerpedjiev *et al.*, 2018). Scaffolds were visually inspected and corrected as described by Howe *et al*. (2021). Manual corrections included four breaks and six joins. The curation process is described at sanger-tol/curation-resources . PretextSnapshot was used to generate a Hi-C contact map of the final assembly.

### Assembly quality assessment

The MerquryFK tool ( Rhie *et al.*, 2020) was run in a Singularity container ( Kurtzer *et al.*, 2017) to evaluate *k*-mer completeness and assembly quality for both haplotypes using the *k*-mer database ( *k* = 31) computed prior to genome assembly. The analysis outputs included assembly QV scores and completeness statistics.

The genome was analysed using the BlobToolKit pipeline, a Nextflow implementation of the earlier Snakemake version ( Challis *et al.*, 2020). PacBio reads were aligned to the assembly using minimap2 ( Li, 2018) and processed with SAMtools ( Danecek *et al.*, 2021) to generate coverage tracks. The pipeline runs BUSCO ( Manni *et al.*, 2021) using lineages identified from the NCBI Taxonomy ( Schoch *et al.*, 2020). For the three basal BUSCO lineages, eukaryota, bacteria and archaea, BUSCO genes are aligned to the UniProt Reference Proteomes database ( Bateman *et al.*, 2023) using DIAMOND BLASTp ( Buchfink *et al.*, 2021). The genome assembly is divided into chunks based on the density of BUSCO genes from the closest taxonomic lineage, and each chunk is aligned to the UniProt Reference Proteomes database using DIAMOND BLASTx. Sequences without DIAMOND BLASTx hits are extracted using seqtk and aligned to the NT database using BLASTn ( Altschul *et al.*, 1990). The outputs are consolidated into a BlobDir for visualisation in BlobToolKit. The BlobToolKit pipeline was developed using nf-core tooling ( Ewels *et al.*, 2020) and MultiQC ( Ewels *et al.*, 2016), with containerisation through Docker ( Merkel, 2014) and Singularity ( Kurtzer *et al.*, 2017).

## Genome sequence report

### Sequence data

**Figure 2.**
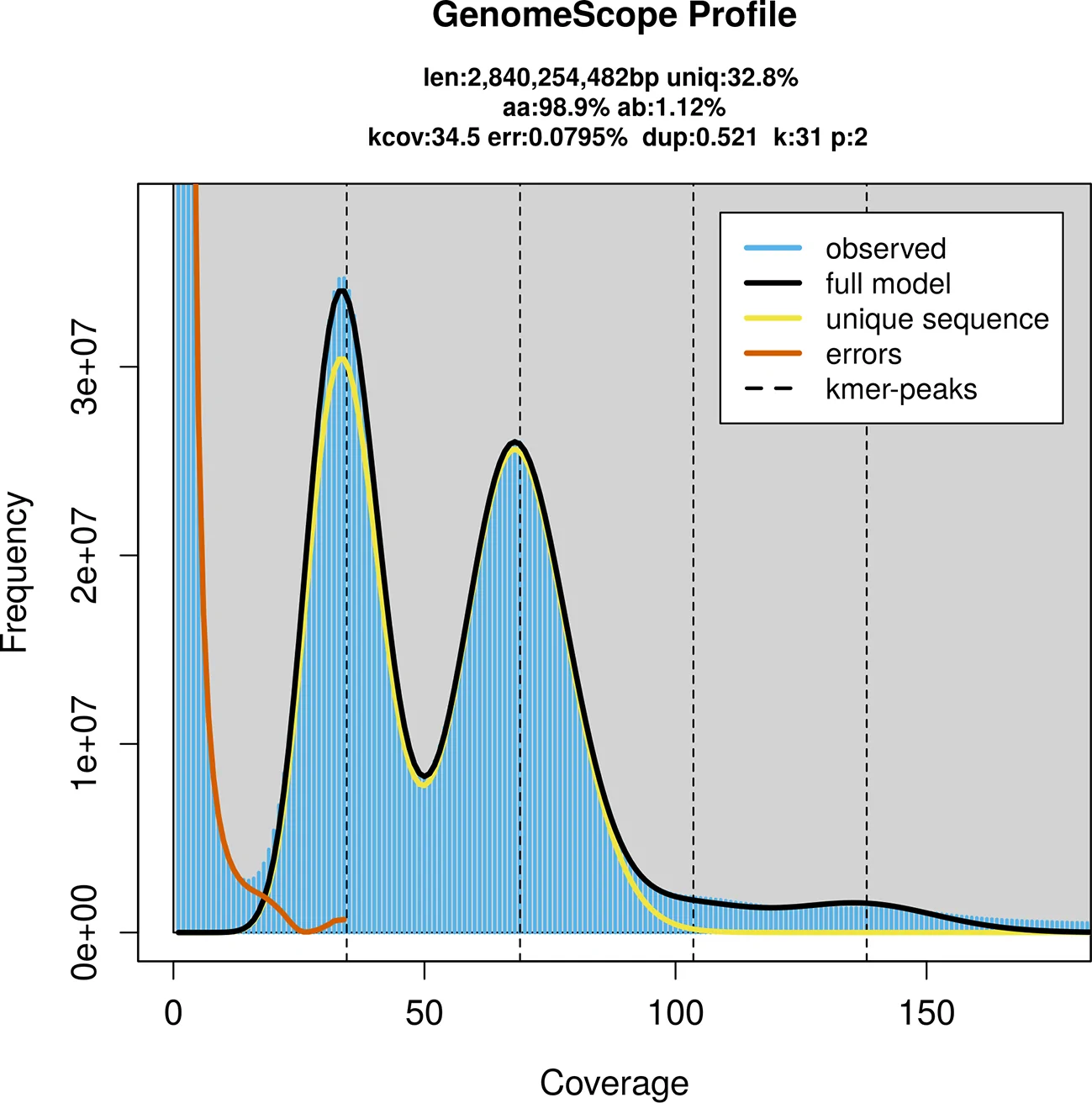
Frequency distribution of *k*-mers generated using GenomeScope2. The plot shows observed and modelled *k*-mer spectra, providing estimates of genome size, heterozygosity, and repeat content based on unassembled sequencing reads.

The genome of a specimen of *Petasites hybridus* was sequenced using Pacific Biosciences single-molecule HiFi long reads, generating 158.98 Gb (gigabases) from 14.72 million reads, which were used to assemble the genome. GenomeScope2.0 analysis using a *p* = 2 model gave a *k*-mer-based genome size estimate of 2 846.13 Mb, with a heterozygosity of 1.12% and repeat content of 67.25% ( Figure 2). Using flow cytometry, the genome size (1C-value) of the sample was estimated to be 3.23 pg, equivalent to 3 160.00 Mb. These estimates guided expectations for the assembly. Based on the estimated genome size, the sequencing data provided approximately 54× coverage. Hi-C sequencing produced 463.71 Gb from 1 535.46 million read pairs, which were used to scaffold the assembly. RNA sequencing data were also generated and are available in public sequence repositories. Table 1 summarises the specimen and sequencing details.

**Table 1.** Specimen and sequencing data for *Petasites hybridus* (BioProject PRJEB80114).

Platform	PacBio HiFi	Hi-C	RNA-seq
**ToLID**	daPetHybr1	daPetHybr1	daPetHybr1
**Specimen ID**	EDTOL03655	EDTOL03655	EDTOL03655
**BioSample (source individual)**	SAMEA110449345	SAMEA110449345	SAMEA110449345
**BioSample (tissue)**	SAMEA110449403	SAMEA110449403	SAMEA110449403
**Tissue**	leaf	leaf	leaf
**Instrument**	Sequel IIe	Illumina NovaSeq X	Illumina NovaSeq X
**Run accessions**	ERR13698297; ERR13698298; ERR13698299	ERR13670060	ERR13670881; ERR14792851
**Read count total**	14.72 million reads	1 535.46 million read pairs	71.74 million read pairs
**Base count total**	158.98 Gb	463.71 Gb	21.67 Gb

### Assembly statistics

The genome was assembled into two haplotypes using Hi-C phasing. Haplotype 1 was curated to chromosome level, while haplotype 2 was assembled to scaffold level. The final assembly has a total length of 2 802.87 Mb in 2 406 scaffolds, with 1 216 gaps, and a scaffold N50 of 90.18 Mb ( Table 2).

**Table 2.** Genome assembly statistics for *Petasites hybridus.*

Genome assembly	Haplotype 1	Haplotype 2
**Assembly name**	daPetHybr1.hap1.1	daPetHybr1.hap2.1
**Assembly accession**	GCA_965123105.1	GCA_965125625.1
**Assembly level**	chromosome	scaffold
**Span (Mb)**	2 802.87	2 756.82
**Number of chromosomes**	30	-
**Number of contigs**	3 622	2 862
**Contig N50**	4.26 Mb	4.5 Mb
**Number of scaffolds**	2 406	1 776
**Scaffold N50**	90.18 Mb	89.12 Mb
**Longest scaffold length (Mb)**	130.11	132.17
**Organelles**	Mitochondrial genome: 302.34 kb; Plastid genome: 150.22 kb	-

The scaffold count follows the NCBI assembly statistics and excludes organelle assemblies, which are reported separately where present.

**Figure 3.**
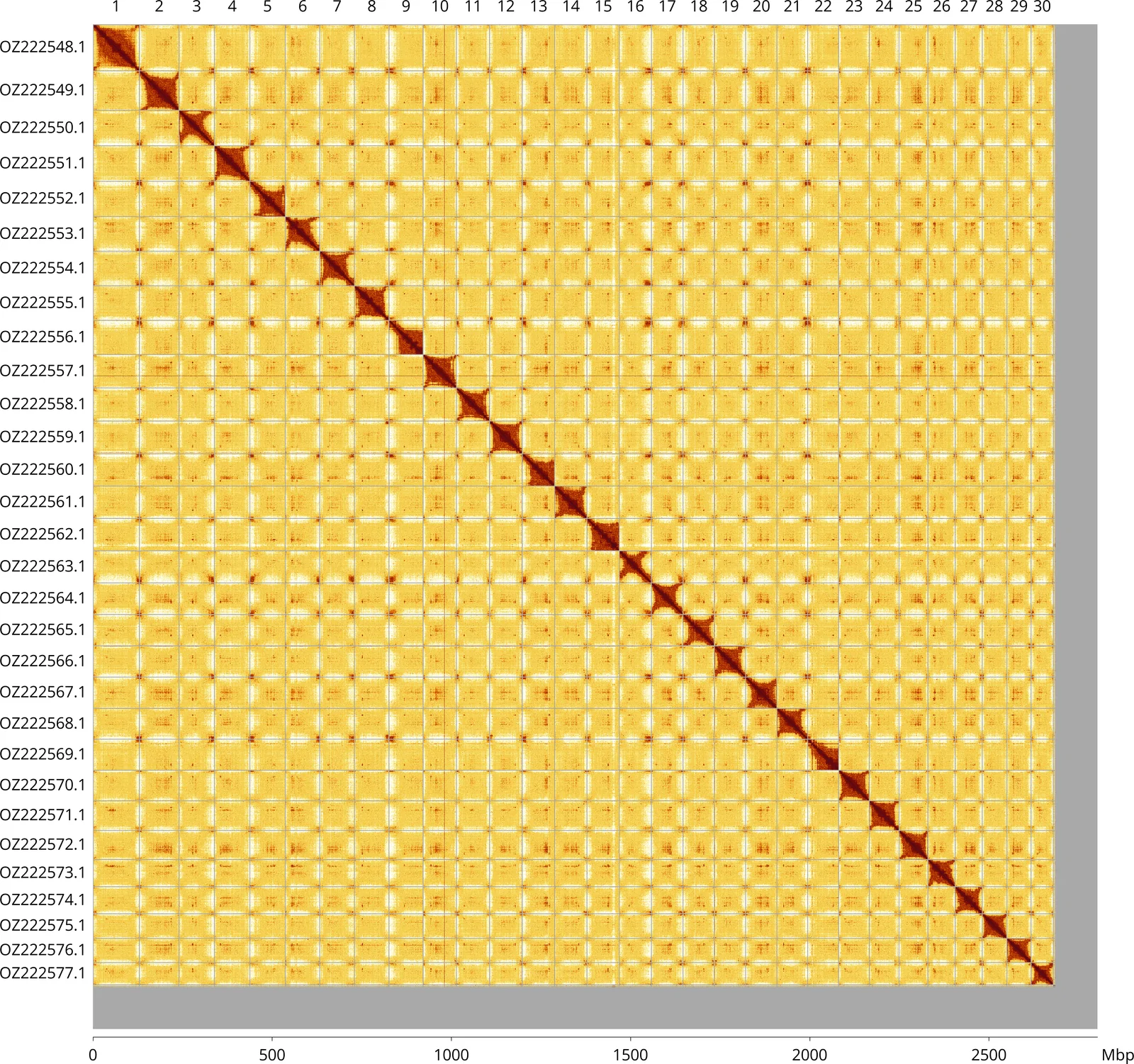
Hi-C contact map of the *Petasites hybridus* genome assembly. Assembled chromosomes are shown in order of size and labelled along the axes, with a megabase scale shown below. The plot was generated using PretextSnapshot.

Most of the assembly sequence (95.61%) was assigned to 30 chromosomal-level scaffolds. These chromosome-level scaffolds, confirmed by Hi-C data, are named according to size ( Figure 3; Table 3). During genome curation, we noticed that there are a large number of heterozygous inversions scattered throughout this genome.

**Table 3.** Chromosomal pseudomolecules in the haplotype 1 genome assembly of *Petasites hybridus* daPetHybr1.

INSDC accession	Molecule	Length (Mb)	GC%
OZ222548.1	1	130.11	35
OZ222549.1	2	109.92	35
OZ222550.1	3	99.20	35
OZ222551.1	4	98.74	35
OZ222552.1	5	98.62	35
OZ222553.1	6	96.79	35
OZ222554.1	7	96.45	35
OZ222555.1	8	96.23	35
OZ222556.1	9	95.13	35
OZ222557.1	10	93.41	35
OZ222558.1	11	92.12	35
OZ222559.1	12	91.31	35
OZ222560.1	13	90.36	35
OZ222561.1	14	90.23	35
OZ222562.1	15	90.18	35.50
OZ222563.1	16	89.62	34.50
OZ222564.1	17	88.34	35
OZ222565.1	18	87.37	35
OZ222566.1	19	87.21	35
OZ222567.1	20	86.96	35
OZ222568.1	21	86.32	35
OZ222569.1	22	86.22	35.50
OZ222570.1	23	85.10	35
OZ222571.1	24	83.34	35
OZ222572.1	25	80.82	35
OZ222573.1	26	75.97	35
OZ222574.1	27	75.09	35
OZ222575.1	28	68.59	35
OZ222576.1	29	67.80	35
OZ222577.1	30	62.25	35

The mitochondrial genome (length 302.34 kb, OZ222578.1) and plastid genome (length 150.22 kb, OZ222579.1) were also assembled. These sequences are included as contigs in the multifasta file of the genome submission and as standalone records.

### Assembly quality metrics

**Figure 4.**
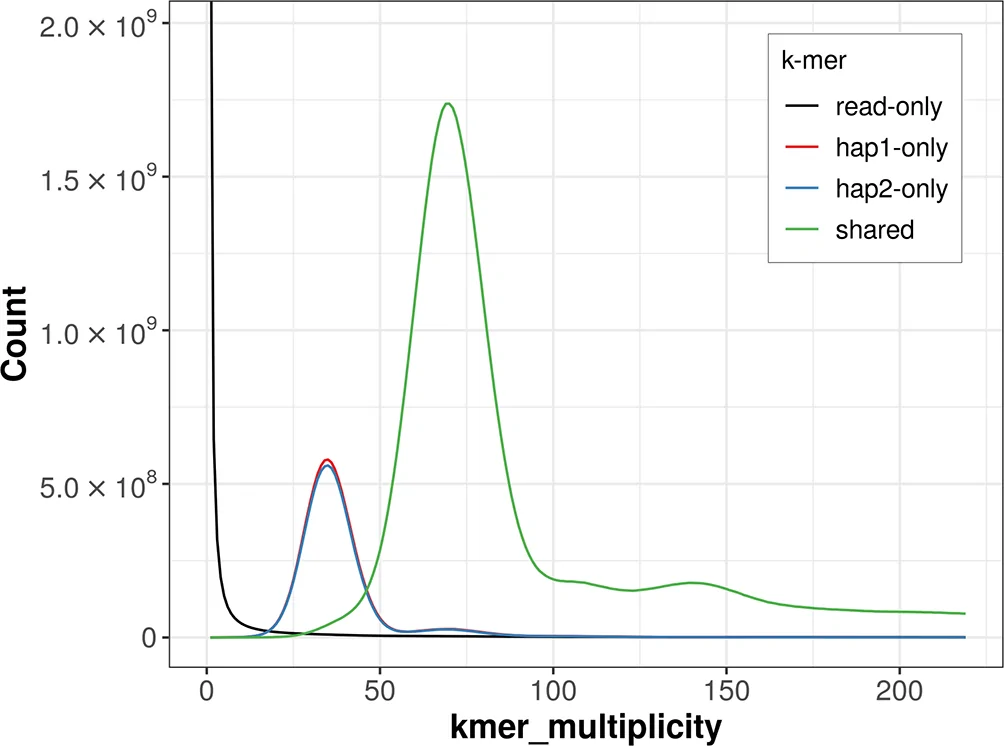
Evaluation of *k*-mer completeness using MerquryFK. This plot illustrates the recovery of *k*-mers from the original read data in the final assemblies. The horizontal axis represents *k*-mer multiplicity, and the vertical axis shows the number of *k*-mers. The black curve represents *k*-mers that appear in the reads but are not assembled. The green curve corresponds to *k*-mers shared by both assemblies, and the red and blue curves show *k*-mers found only in one assembly.

For haplotype 1, the estimated QV is 59.4, and for haplotype 2, 59.5. When the two haplotypes are combined, the assembly achieves an estimated QV of 59.4. The *k*-mer completeness is 79.03% for haplotype 1, 78.33% for haplotype 2, and 98.66% for the combined haplotypes ( Figure 4).

**Figure 5.**
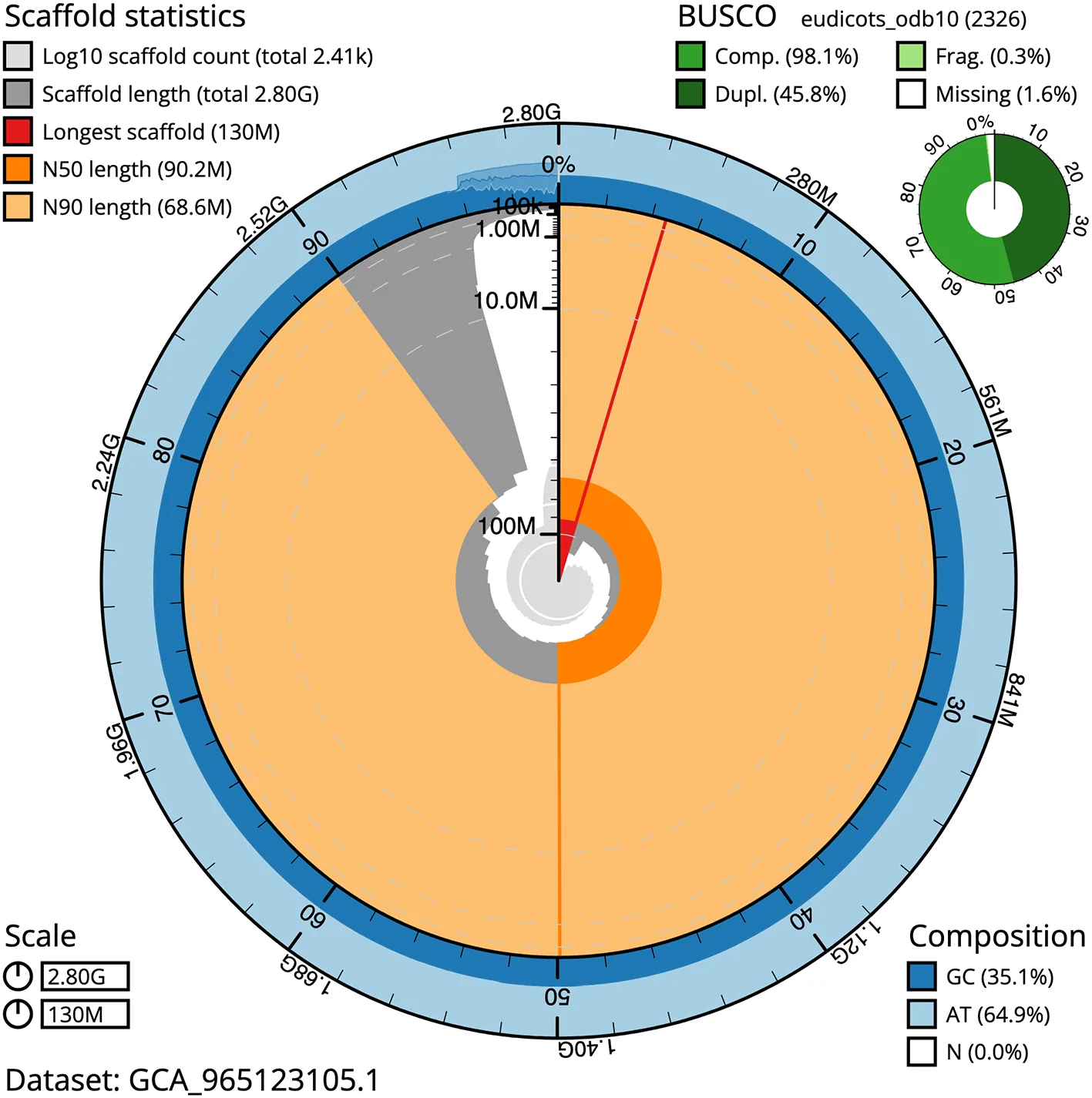
Assembly metrics for daPetHybr1.hap1.1. The BlobToolKit snail plot provides an overview of assembly metrics and BUSCO gene completeness. The circumference represents the length of the whole genome sequence, and the main plot is divided into 1 000 bins around the circumference. The outermost blue tracks display the distribution of GC, AT, and N percentages across the bins. Scaffolds are arranged clockwise from longest to shortest and are depicted in dark grey. The longest scaffold is indicated by the red arc, and the deeper orange and pale orange arcs represent the N50 and N90 lengths. A light grey spiral at the centre shows the cumulative scaffold count on a logarithmic scale. A summary of complete, fragmented, duplicated, and missing BUSCO genes in the eudicots_odb10 set is presented at the top right. An interactive version of this figure can be accessed on the BlobToolKit viewer.

**Figure 6.**
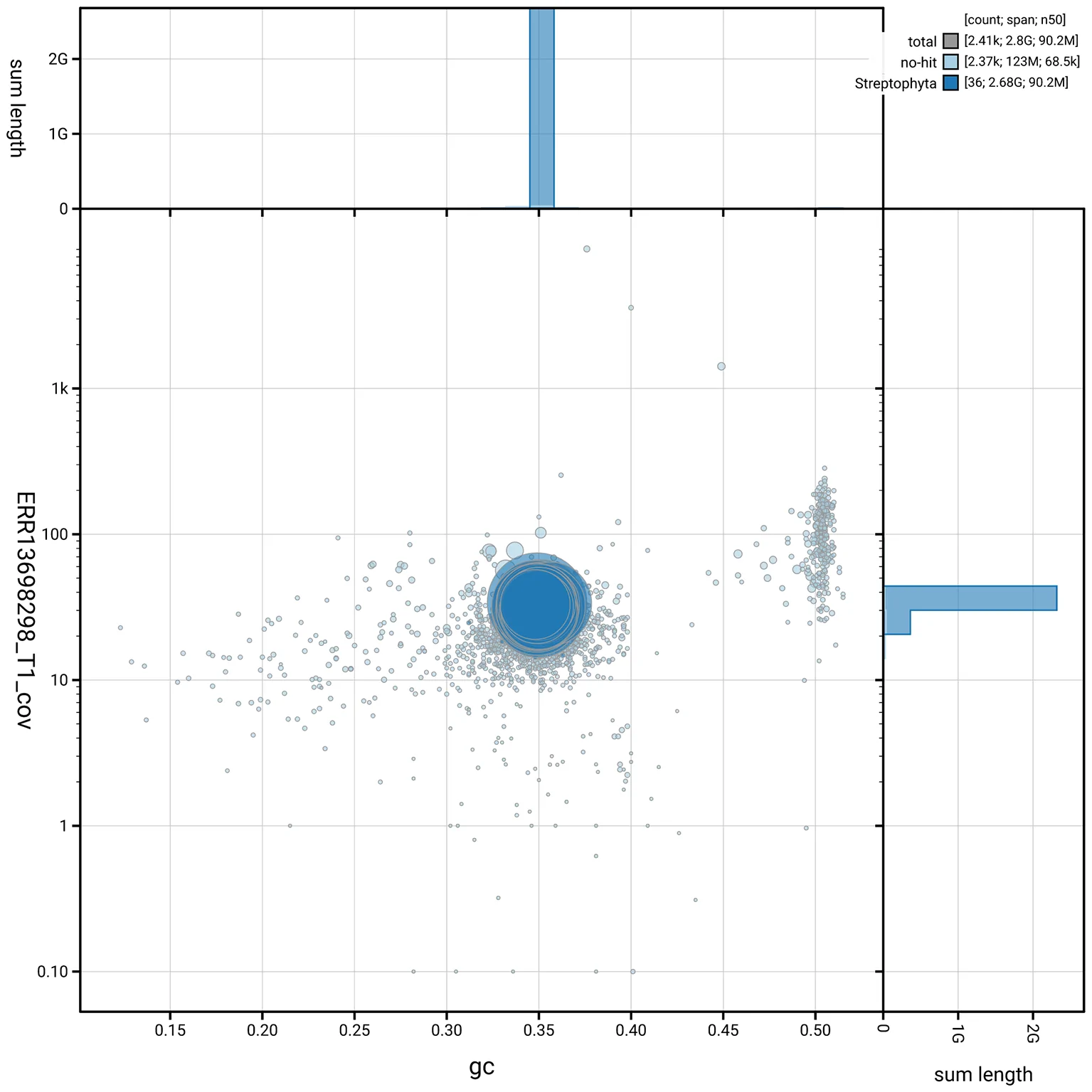
BlobToolKit blob plot for daPetHybr1.hap1.1. The plot shows base coverage (vertical axis) and GC content (horizontal axis). The circles represent scaffolds, with the size proportional to scaffold length and the colour representing phylum membership. The histograms along the axes display the total length of sequences distributed across different levels of coverage and GC content. An interactive version of this figure is available on the BlobToolKit viewer.

BUSCO analysis using the eudicots_odb10 reference set ( *n* = 2 326) identified 98.1% of the expected gene set (single = 52.3%, duplicated = 45.8%) for haplotype 1. The snail plot in Figure 5 summarises the scaffold length distribution and other assembly statistics for haplotype 1. The blob plot in Figure 6 shows the distribution of scaffolds by GC proportion and coverage for haplotype 1.

Table 4 lists the assembly metric benchmarks adapted from Rhie *et al.* (2021) and the Earth BioGenome Project Report on Assembly Standards January 2026. The EBP metric calculated for haplotype 1 is **6.C.Q59**, meeting the recommended 6.C.Q40 reference standard.

**Table 4.** Earth Biogenome Project summary metrics for the *Petasites hybridus* assembly.

Measure	Value	Benchmark
EBP summary (haplotype 1)	6.C.Q59	6.C.Q40
Contig N50 length	4.26 Mb	≥ 1 Mb
Scaffold N50 length	90.18 Mb	= chromosome N50
Consensus quality (QV)	Haplotype 1: 59.4; haplotype 2: 59.5; combined: 59.4	≥ 40
*k*-mer completeness	Haplotype 1: 79.03%; Haplotype 2: 78.33%; combined: 98.66%	≥ 90%
BUSCO	Haplotype 1: C:98.1% [S:52.3%, D:45.8%], F:0.3%, M:1.6%, n:2 326; Haplotype 2: C:97.9% [S:52.5%, D:45.4%], F:0.3%, M:1.8%, n:2 326	S > 90%; D < 5%
Percentage of assembly assigned to chromosomes	95.61%	≥ 90%

**Notes:** The EBP summary uses log10(Contig N50); chromosome-level (C) or log10(Scaffold N50); Q (Merqury QV). BUSCO: C = complete; S = single-copy; D = duplicated; F = fragmented; M = missing; n = orthologues.

**Table 5.** Software versions and sources used for *Petasites hybridus.*

Software	Version	Source
BLAST	2.14.0	ftp://ftp.ncbi.nlm.nih.gov/blast/executables/blast+/
BlobToolKit	4.3.9	https://github.com/blobtoolkit/blobtoolkit
BUSCO	5.5.0	https://gitlab.com/ezlab/busco
bwa-mem2	2.2.1	https://github.com/bwa-mem2/bwa-mem2
DIAMOND	2.1.8	https://github.com/bbuchfink/diamond
fasta_windows	0.2.4	https://github.com/tolkit/fasta_windows
FastK	1.1	https://github.com/thegenemyers/FASTK
GenomeScope2.0	2.0.1	https://github.com/tbenavi1/genomescope2.0
Gfastats	1.3.6	https://github.com/vgl-hub/gfastats
Hifiasm	0.19.8-r603	https://github.com/chhylp123/hifiasm
HiGlass	1.13.4	https://github.com/higlass/higlass
MerquryFK	1.1.0-c1	https://github.com/thegenemyers/MERQURY.FK
Minimap2	2.24-r1122	https://github.com/lh3/minimap2
Oatk	0.9	https://github.com/c-zhou/oatk
MultiQC	1.14; 1.17 and 1.18	https://github.com/MultiQC/MultiQC
Nextflow	23.10.0	https://github.com/nextflow-io/nextflow
PretextSnapshot	0.0.5	https://github.com/sanger-tol/PretextSnapshot
PretextView	1.0.3	https://github.com/sanger-tol/PretextView
samtools	1.19.2	https://github.com/samtools/samtools
sanger-tol/ascc	0.1.0	https://github.com/sanger-tol/ascc
sanger-tol/blobtoolkit	0.6.0	https://github.com/sanger-tol/blobtoolkit
sanger-tol/curationpretext	1.4.2	https://github.com/sanger-tol/curationpretext
Seqtk	1.3	https://github.com/lh3/seqtk
Singularity	3.9.0	https://github.com/sylabs/singularity
TreeVal	1.4.0	https://github.com/sanger-tol/treeval
YaHS	1.2a.2	https://github.com/c-zhou/yahs

### Genome annotation report

The *Petasites hybridus* genome assembly (GCA_965123105.1) was annotated by Ensembl at the European Bioinformatics Institute (EBI). This annotation includes 95 872 transcribed mRNAs from 41 187 protein-coding and 40 426 non-coding genes. The average transcript length is 2 253.07 bp, with an average of 1.17 coding transcripts per gene and 3.85 exons per transcript. For further information about the annotation, please refer to the annotation page on Ensembl.

## Author information

• Members of the Royal Botanic Garden Edinburgh Genome Acquisition Lab
• Members of the Plant Genome Sizing Collective
• Members of the Darwin Tree of Life Barcoding collective
• Members of the Wellcome Sanger Institute Tree of Life Management, Samples and Laboratory team
• Members of Wellcome Sanger Institute Scientific Operations – Sequencing Operations
• Members of the Wellcome Sanger Institute Tree of Life Core Informatics team
• Members of the Tree of Life Core Informatics collective
• Members of the Darwin Tree of Life Consortium

## Wellcome sanger institute – Legal and governance

The materials that have contributed to this genome note have been supplied by a Darwin Tree of Life Partner. The submission of materials by a Darwin Tree of Life Partner is subject to the **‘Darwin Tree of Life Project Sampling Code of Practice’**, which can be found in full on the Darwin Tree of Life website. By agreeing with and signing up to the Sampling Code of Practice, the Darwin Tree of Life Partner agrees they will meet the legal and ethical requirements and standards set out within this document in respect of all samples acquired for, and supplied to, the Darwin Tree of Life Project. Further, the Wellcome Sanger Institute employs a process whereby due diligence is carried out proportionate to the nature of the materials themselves, and the circumstances under which they have been/are to be collected and provided for use. The purpose of this is to address and mitigate any potential legal and/or ethical implications of receipt and use of the materials as part of the research project, and to ensure that in doing so we align with best practice wherever possible. The overarching areas of consideration are:

• Ethical review of provenance and sourcing of the material
• Legality of collection, transfer and use (national and international)

Each transfer of samples is further undertaken according to a Research Collaboration Agreement or Material Transfer Agreement entered into by the Darwin Tree of Life Partner, Genome Research Limited (operating as the Wellcome Sanger Institute), and in some circumstances, other Darwin Tree of Life collaborators.

## Data Availability

European Nucleotide Archive: Petasites hybridus. Accession number PRJEB80114; https://identifiers.org/ena.embl/PRJEB80114. The genome sequence is released openly for reuse. The *Petasites hybridus* genome sequencing initiative is part of the Darwin Tree of Life Project (PRJEB40665) and the Sanger Institute Tree of Life Programme (PRJEB43745). All raw sequence data and the assembly have been deposited in INSDC databases. Raw data and assembly accession identifiers are reported in Tables 1 and 2. Pipelines used for genome assembly at the WSI Tree of Life are available at https://pipelines.tol.sanger.ac.uk/pipelines. Table 5 lists software versions used in this study.
