# Supplementary material for: The genome sequence of Petasites hybridus (L. G.Gaertn., B.Mey. & Scherb., 1802) (Asterales: Asteraceae)
Source: Wellcome Open Res. 2026 Jul 29;11:479. [Version 1] doi: 10.12688/wellcomeopenres.27231.1 (PMC13586604; doi:10.12688/wellcomeopenres.27231.1)
Supplement: Peer review history

# Reviewer report: Reviewer response for version 1

**DOI:** 10.21956/wellcomeopenres.29971.r166818

**Published:** 2026-09-17

**Authors:** Rong Zhang

**Affiliations:** 1 Kunming Institute of Botany, Kunming, Yunnan, China

This genome note reports a high-quality chromosome-level assembly of *Petasites hybridus*. The contig-level QV and other core assembly statistics are excellent considering the large genome size of this species. Overall, the work is suitable for indexing after minor revisions. Its better to include LAI assessment for the chromosome assembly. De novo gene prediction for hap2 is not required. However, it is recommended to lift gene models from hap1 to hap2 using Liftoff and report the number of successfully lifted genes in supplementary material. This simple analysis provides useful information about gene content conservation between the two haplotypes.

Are sufficient details of methods and materials provided to allow replication by others?

Yes

Is the rationale for creating the dataset(s) clearly described?

Yes

Are the datasets clearly presented in a useable and accessible format?

Yes

Are the protocols appropriate and is the work technically sound?

Yes

Reviewer Expertise:

My research focuses on the phylogeny, phylogenomics and evolution; I think the assembly quality reported in this manuscript is comparable to that of other DToL genome assemblies.

I confirm that I have read this submission and believe that I have an appropriate level of expertise to confirm that it is of an acceptable scientific standard.

# Reviewer report: Reviewer response for version 1

**DOI:** 10.21956/wellcomeopenres.29971.r166821

**Published:** 2026-09-08

**Authors:** Matthew W. Davis

**Affiliations:** 1 University of California, California, USA

**Article summary:**

 The article reports a genome assembly of *Petastites hybridius*, a plant in Asteraceae that has historically been used medicinally and has very interesting floral structure. The authors utilize the current best practices for data generation and genome assembly, while the manuscript complies with the general Tree of Life format. Overall, the assembled genome appears to be of high quality, and there are minor comments to address.

**Reviewer comments:**

 There is a very high duplicated BUSCO score of 45.8%. This could be indicative of polyploidy, however there is no mention of ploidy in the manuscript. The authors should make a comment on this.

 Smudgeplot (Ranallo-Benavidez et al., 2020) could be added to the general list of analyses done on every genome assembly by the Tree of Life project to try and detect putative instances of polyploidy. As it is a part of the GenomeScope2 toolkit family, it should be amenable to the system being implemented now and not add too much additional computational strain or complexity that would meaningfully impede the larger goal of the project. This validation would help further benefit the community.

**Lines with comments and questions:**

 “...the relevant DNA barcode region was also used alongside the initial barcoding data for sample tracking at the WSI (Twyford et al., 2024).”

- Wellcome Sanger Institute should be fully written here, as it is the first mention of the abbreviation.

**Questions for authors:**

 When referring to scaffold level and chromosome level assemblies, are the authors using the NCBI definitions?

 From the NCBI glossary:

- Chromosome

There is sequence for one or more chromosomes. This may be a completely sequenced chromosome without gaps or a chromosome containing spans with gaps between them. There may also be unplaced or unlocalized sequences.

- Scaffold

One or more contigs are connected across gaps of 10 or more bases to create scaffolds. All sequences are unplaced or unlocalized.

 From the scaffolding methods, it seems both haplotypes are scaffolded with Hi-C, so is the terminology just to imply lower confidence in the haplotype 2 assembly?

**Final Reviewer thoughts:**

 For the goal of the larger Tree of Life project, the assembly is of high quality and will provide a valuable resource for the conservation, evolution, and population biology communities. There is some work necessary to further validate the ploidy of the species, and additional clarification of assembly quality classification would be helpful, but the current state of the genome for use as a reference seems accurate and usable.

Are sufficient details of methods and materials provided to allow replication by others?

Yes

Is the rationale for creating the dataset(s) clearly described?

Yes

Are the datasets clearly presented in a useable and accessible format?

Yes

Are the protocols appropriate and is the work technically sound?

Yes

Reviewer Expertise:

Plant biology, genomics, mutation biology

I confirm that I have read this submission and believe that I have an appropriate level of expertise to confirm that it is of an acceptable scientific standard.
